# Development of a predictive nomogram for in-hospital death risk in multimorbid patients with hepatocellular carcinoma undergoing Palliative Locoregional Therapy

**DOI:** 10.1038/s41598-024-64457-y

**Published:** 2024-06-17

**Authors:** Rucheng Yao, Bowen Zheng, Xueying Hu, Baohua Ma, Jun Zheng, Kecheng Yao

**Affiliations:** 1 Department of Hepatopancreatobilary Surgery, The First College of Clinical Medical Science, Three Gorges University, Yichang, Hubei China; 2 Department of Geriatrics, The First College of Clinical Medical Science, Three Gorges University, Yichang, Hubei China; 3 Department of Medical Record, The First College of Clinical Medical Science, Three Gorges University, Yichang, Hubei China; 4 The People’s Hospital of China Three Gorges University, Yichang, Hubei China; 5 Yichang Central People’s Hospital, Yichang, Hubei China

**Keywords:** Hepatocellular carcinoma, Multimorbidity, In-hospital mortality, Nomogram, Predictive model, Cancer epidemiology, Outcomes research, Risk factors, Cancer epidemiology

## Abstract

Patients diagnosed with hepatocellular carcinoma (HCC) often present with multimorbidity, significantly contributing to adverse outcomes, particularly in-hospital mortality. This study aimed to develop a predictive nomogram to assess the impact of comorbidities on in-hospital mortality risk in HCC patients undergoing palliative locoregional therapy. We retrospectively analyzed data from 345 hospitalized HCC patients who underwent palliative locoregional therapy between January 2015 and December 2022. The nomogram was constructed using independent risk factors such as length of stay (LOS), hepatitis B virus (HBV) infection, hypertension, chronic obstructive pulmonary disease (COPD), anemia, thrombocytopenia, liver cirrhosis, hepatic encephalopathy (HE), N stage, and microvascular invasion. The model demonstrated high predictive accuracy with an AUC of 0.908 (95% CI: 0.859–0.956) for the overall dataset, 0.926 (95% CI: 0.883–0.968) for the training set, and 0.862 (95% CI: 0.728–0.994) for the validation set. Calibration curves indicated a strong correlation between predicted and observed outcomes, validated by statistical tests. Decision curve analysis (DCA) and clinical impact curves (CIC) confirmed the model's clinical utility in predicting in-hospital mortality. This nomogram offers a practical tool for personalized risk assessment in HCC patients undergoing palliative locoregional therapy, facilitating informed clinical decision-making and improving patient management.

## Introduction

Primary liver cancer, predominantly hepatocellular carcinoma (HCC), is a leading malignancy worldwide^1^. In 2020, China reported 410,038 new liver cancer cases and 391,152 deaths, accounting for nearly half of the global incidence and mortality^2^. Approximately 70% of HCC patients in China are diagnosed at an advanced stage, resulting in a high recurrence rate within five years post-surgery^2,3^. In-hospital mortality for HCC patients varies significantly from 2.2% to 25.3%, influenced by economic status, tumor burden, and comorbidities^4–7^.

The etiology of HCC includes chronic hepatitis B and C infections, alcohol consumption, and nonalcoholic steatohepatitis, all leading to complex clinical presentations and prognoses. Managing HCC is particularly challenging in patients with multiple comorbidities such as hypertension, diabetes, COPD, and renal failure, which significantly increase the risk of in-hospital mortality^8,9^.

Currently, nomograms are emerging as powerful predictive tools in oncology^10,11^, offering personalized risk assessments to enhance clinical decision-making and patient counselling^12–14^. In various cancers, nomograms have been successfully employed to provide personalized risk assessments, enhancing clinical decision-making and patient counselling. However, existing tools often lack specificity for predicting short-term mortality risk in HCC patients undergoing interventional therapy.

The implementation of localized treatment strategies such as transarterial chemoembolization (TACE), radiotherapy (RT), and radiofrequency ablation (RFA) has notably contributed to a reduction in mortality rates and an extension of survival duration in HCC patients^13,15–17^. Despite these advancements, the management of HCC patients with multiple comorbidities remains complex, necessitating a precise risk assessment tool.

To address this need, we developed a predictive nomogram that integrates the impact of comorbidities on in-hospital mortality. This tool aims to improve individualized patient care by providing accurate mortality risk predictions, facilitating better clinical decision-making and resource allocation.

## Methods

### Purpose

In clinical practice, it is common to encounter HCC patients who are ineligible for curative surgery due to their multimorbidity and tumor burden. We aim to understand the comorbid conditions and their association with in-hospital mortality among such patients who opt for palliative interventional treatments. This cross-sectional study explores the multimorbidity correlations with in-hospital mortality and develops a nomogram to predict the risk of in-hospital death in HCC patients undergoing interventional therapy.

### Study design and data source

The study protocol was approved by the Ethics Committee of Yichang Central People's Hospital (Approval No: 2023–1101). Due to the retrospective cross-sectional design of the study, the requirement for informed consent was waived. All methods were conducted in accordance with the relevant guidelines and regulations of Yichang Central People's Hospital. This retrospective cross-sectional study included patient data sourced from the Yichang Central People’s Hospital over a period extending between January 2015 and December 2022, retrieved from a comprehensive hospital-based electronic database. All data were collected at the point of the patient’s admission. As this is a cross-sectional study, data collection occurred at a single time point only, with no follow-up, hence there are no censored patients. The article has been structured according to the TRIPOD guidelines to ensure clarity and transparency in reporting our prognostic study.

### Participants

Eligibility criteria included: (1) presence of comorbidities at admission; (2) primary diagnosis upon admission; and (3) classification of the main diagnosis according to the International Classification of Diseases for Oncology (ICD-O) codes (M8170/3, M8171/3, M8172/3, M8173/3, M8174/3, and M8175/3) alongside the International Classification of Diseases (10th edition) codes (C22.000). The inclusion criteria were as follows: (1) a definitive diagnosis of HCC at discharge coupled with a minimum hospitalization period of 24 h and (2) the administration of one of three treatment modalities during hospitalization—TACE, RT, or RFA. The exclusion criteria for patients were as follows: (1) essential information was unavailable; (2) the patient required admission to the intensive care unit; and (3) an absence of prior medical history data. The data collected were demographic data, patient comorbidities, and treatment outcomes, and they were extracted from electronic medical records.

### Outcome

The primary outcome of interest is in-hospital mortality, defined as death occurring during hospitalization, which is coded based on the patient's discharge disposition (alive or deceased). We selected variables associated with in-hospital mortality that are available from the electronic medical record database. These include gender, age, readmissions, smoking history, alcohol consumption history, medical history, TNM stage, tumor characteristics, and modes of interventional treatment (e.g., TACE, RT, RFA).

### Predictors

The predictors used in developing the nomogram were clearly defined and included demographic variables (gender, age), clinical parameters (length of stay (LOS), smoking history, alcohol consumption history), comorbidities (HBV status, hypertension, COPD, asthma, CKD, anemia, thrombopenia, ascites, pneumonia, liver cirrhosis, hepatic encephalopathy (HE)), and tumor characteristics (TNM stage, portal vein invasion, microvascular invasion). All variables were collected from the electronic medical record system. Comorbidities were identified based on the medical history at admission and discharge diagnoses. If a patient had a documented history of a chronic disease or if the discharge diagnosis included the chronic disease, the corresponding variable was coded as 1. If there was no report or discharge diagnosis of the disease, the variable was coded as 0. Tumor characteristics were similarly coded: the presence of specific tumor-related conditions such as portal vein invasion or microvascular invasion was assigned a value of 1, and their absence was assigned a value of 0. This method ensured a comprehensive and standardized collection of predictor variables, reflecting the true clinical status of each patient.

We used CT or MRI imaging techniques to indirectly assess microvascular invasion (MVI) in liver cancer patients^18^. All imaging data were sourced from our hospital's imaging database, including contrast-enhanced CT and enhanced MRI. During image loading and preprocessing, two experienced radiologists independently evaluated the imaging features, ensuring they were blinded to the patients' clinical information. The evaluated imaging features included nonsmooth tumor margins, peritumoral enhancement, and TTPVI (Two-Trait Predictor of Venous Invasion), which comprises internal arteries and a low-density halo (or low-signal halo). The presence and combination of these features were used to predict the likelihood of MVI, thereby supporting clinical decision-making.

To reduce bias, the assessment of predictors was blinded to the outcome of in-hospital mortality, and vice versa. The clinical staff responsible for recording the patients' medical history and comorbidities were unaware of the study's outcome measurements, which were determined independently. Additionally, those analyzing the data and building the predictive models were blinded to the patients' clinical characteristics during the initial data collection phase. This ensured that the predictor variables were assessed independently of the outcomes, thus minimizing potential biases in the data analysis.

The candidate variables for our prognostic analysis were chosen based on their availability in the hospital records and their clinical relevance to hepatocellular carcinoma (HCC) patient outcomes. These variables are detailed in Table 1.

**Table 1:** Baseline data of patients with HCC following interventional therapy.

Characteristics	Patients		In-hospital mortality		P-value
	n	%	n	%	
Gender					0.371
Male	277	80.3	30	10.8	
Female	68	19.7	10	14.7	
Age					0.883
< 65	186	53.9	22	11.8	
≥ 65	159	46.1	18	11.3	
Readmission					0.697
Yes	242	70.1	27	11.2	
No	103	29.9	13	12.6	
Smoking					0.873
Yes	116	33.6	13	11.2	
No	229	66.4	27	11.8	
Alcohol					0.002
Yes	115	33.3	22	19.1	
No	230	66.7	18	7.8	
Hepatitis B virus					0.012
Yes	160	46.4	26	16.3	
No	185	53.6	14	7.6	
Hypertension					0.005
Yes	161	46.7	27	16.8	
No	184	53.3	13	7.1	
CHD					0.921
Yes	67	19.4	8	11.9	
No	278	80.6	32	11.5	
Heart Failure					0.024
Yes	53	15.4	11	20.8	
No	292	84.6	29	9.9	
TIA					0.028
Yes	151	43.8	24	15.9	
No	194	56.2	16	8.2	
COPD					0.003
Yes	140	40.6	25	17.9	
No	205	59.4	15	7.3	
Asthma					0.002
Yes	13	3.8	5	38.5	
No	332	96.2	35	10.5	
Diabetes					0.031
Yes	68	19.7	13	19.1	
No	277	80.3	27	9.7	
CKD					0.043
Yes	147	42.6	23	15.6	
No	198	57.4	17	8.6	
Anemia					0.004
Yes	39	11.3	10	25.6	
No	306	88.7	30	9.8	
Thrombopenia					0.016
Yes	130	37.7	22	16.9	
No	215	62.3	18	8.4	
Ascites					0.026
Yes	126	36.5	21	16.7	
No	219	63.5	19	8.7	
Pneumonia					0.011
Yes	151	43.8	25	16.6	
No	194	56.2	15	7.7	
Liver cirrhosis					0.001
Yes	110	31.9	22	20.0	
No	235	68.1	18	7.7	
HE					0.010
Yes	111	32.2	20	18.0	
No	234	67.8	20	8.5	
T Stage					0.001
T1	39	11.3	1	2.6	
T2	46	13.3	1	2.2	
T3	53	15.4	2	3.8	
T4	207	60.0	36	17.4	
N Stage					0.001
N1	128	37.1	24	18.8	
N0	217	62.9	16	7.4	
M Stage					0.063
M1	66	19.1	12	18.2	
M0	279	80.9	28	10.0	
Portal Vein invasion					0.002
Yes	144	41.7	26	18.1	
No	201	58.3	14	7.0	
Microvascular invasion					0.003
Yes	183	53.0	30	16.4	
No	162	47.0	10	6.2	
Therapy Type					0.664
TACE	196	56.8	22	11.2	
RT	112	32.5	15	13.4	
RFA	37	10.7	3	8.1	

*HE* Hepatic Encephalopathy, *TACE* Transhepatic arterial chemoembolization. *RT* Radioactive Therapy, *RFA* Radiofrequency Ablation.

### Sample size

Currently, the academic community lacks a unified approach for deriving risk prediction models and determining requisite sample sizes for validation studies. In the context of model derivation, a general recommendation posits the necessity of a minimum of 10 events per candidate variable^19^. In our study, we incorporated 29 candidate variables within the framework of a binary logistic regression model analysis. Adhering to these guidelines, the minimal sample size for robust validation was calculated to be at least 290 individuals^19^.

### Missing data

In this study, missing data were present in two variables: AFP and CEA, with missing rates of 5.8% and 10.4%, respectively. To ensure the completeness and reliability of the statistical analysis, we used multiple imputation to handle the missing data, specifically employing the predictive mean matching (PMM) method. The imputation process was implemented in R using the "mice" package and the "mice" function for imputation. While we aimed to include all relevant prognostic variables used in clinical practice, certain liver function tests (e.g., ALT, AST, TBIL, albumin) had more than 20% missing values. This high rate of missing data was primarily due to variations in clinical practice and incomplete documentation in medical records. As a result, we were unable to use multiple imputation methods to address these missing values without risking significant bias in our analysis. We ensured that all other available and relevant data were included to build a robust prognostic model.

### Statistical analysis methods

In this study, a total of 345 patients were included in the overall dataset and randomly divided into a training cohort of 242 patients and a validation cohort of 103 patients in a 7:3 ratio. Statistical analysis was conducted using R software (version 3.6.3; available at https://www.R-project.org) and SPSS version 25.0 (IBM, Armonk, NY, USA). We expressed categorical variables as frequencies (percentages) and continuous variables as means (standard deviations). To evaluate differences in baseline characteristics, we used Student's t-test or the nonparametric Mann–Whitney U test for continuous variables, and the Chi-square test or Fisher's exact test for categorical variables. In addition to comparing differences between the mortality group and the overall dataset, we also compared categorical variables between the overall set, training set, and validation set using the Chi-square test or Fisher's exact test, and continuous variables using one-way ANOVA or the Kruskal–Wallis test, as appropriate.

The 'glmnet' package facilitated the identification of factors influencing the risk of in-hospital mortality using the least absolute shrinkage and selection operator (LASSO) method, which is preferred over univariate logistic regression for variable selection in predictive modeling. LASSO was chosen for its ability to handle high-dimensional data and multicollinearity by shrinking some coefficients to zero, thus performing variable selection and regularization simultaneously. We selected the optimal lambda (λ) value with the minimal standard error through cross-validation.

Multivariate logistic regression analysis was used to identify independent clinical predictors associated with in-hospital mortality, considering P values < 0.05 to be significant. A nomogram based on these multivariate risk factors was created to predict in-hospital mortality. The nomogram's predictive accuracy was evaluated using the area under the receiver operating characteristic curve (AUC-ROC), and calibration curves were generated to align the predicted probabilities with the observed probabilities.

For validation, a bootstrap method with 1,000 resamples was applied to the in-hospital mortality nomogram, yielding a bias-corrected C-index. Decision curve analysis (DCA) was used to assess the clinical utility of the in-hospital mortality nomogram by quantifying the net benefits at various threshold probabilities in the study cohort. Net benefits were computed by offsetting the proportion of false positives against true positives while considering the relative harms of foregoing intervention against those of unnecessary intervention.

### Risk groups

Details on how risk groups were created were based on the stratification of predicted probabilities derived from the nomogram. High-risk groups were defined as patients with predicted probabilities above a certain threshold, while low-risk groups had probabilities below that threshold. To determine the optimal threshold for defining high-risk and low-risk groups, we used a combination of clinical relevance and statistical methods. Initially, the Youden Index, which maximizes the sum of sensitivity and specificity, was calculated using the training dataset to identify the threshold that provides the best balance between true positive and false positive rates. This threshold was then validated using the testing dataset to ensure its robustness and clinical relevance. The threshold for defining high-risk and low-risk groups was ultimately set at 0.20, based on these considerations.

### Development vs. validation

In the development phase, the model was constructed using the training dataset. The nomogram was developed based on multivariate logistic regression analysis, incorporating predictors identified through LASSO regression. The model's performance was assessed using the training data. In the validation phase, the model's performance was evaluated using the validation dataset. Differences between the development and validation datasets were assessed in terms of setting, eligibility criteria, outcome, and predictors. This step ensured the model's robustness and generalizability across different patient populations and clinical settings.

### Ethics statement

For the publication of any potentially identifiable images or data included in this article, written informed consent was duly obtained from the respective individual(s). This procedure adheres to ethical standards for the protection of personal privacy and aligns with the guidelines for the dissemination of identifiable research data.

## Results

### Patient characteristics

**Figure 1:**
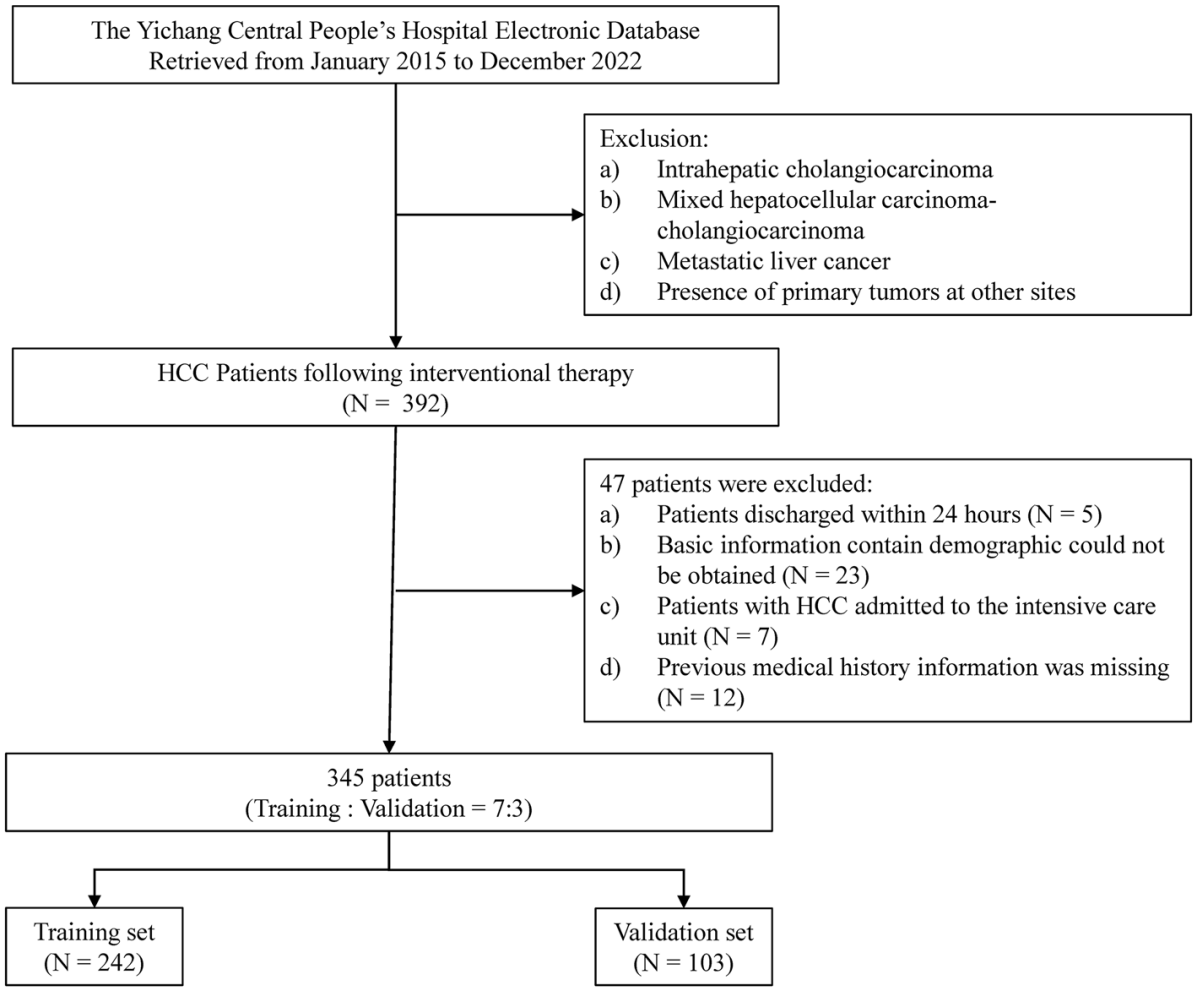
Flow diagram of the selection of eligible patients.

From January 2015 to December 2022, 392 HCC patients underwent interventional therapy. After applying exclusion criteria, 345 patients were eligible for analysis (Fig. 1). The average age of patients in the survival and the in-hospital death groups were 62.25 ± 11.68 and 62.10 ± 11.39 years, respectively (p = 0.010). The mean LOS for these groups were 15.48 ± 4.71 and 17.53 ± 4.81 days, respectively (p = 0.938). The average AFP levels were 354.67 ± 160.71 ng/ml and 358.76 ± 169.73 ng/ml, respectively (p = 0.851). CEA levels were 4.48 ± 1.07 ng/ml and 4.71 ± 1.07 ng/ml, respectively (p = 0.205). In-hospital mortality was 11.6% (40 patients). The cohort was split into a training set (214 patients, 28 deaths) and a validation set (91 patients, 12 deaths).

The patients’ comorbidities included hepatitis B virus (HBV) infection (160 patients, 46.4%), hypertension (161 patients, 46.7%), coronary heart disease (CHD, 67 patients, 19.4%), heart failure (53 patients, 15.4%), chronic obstructive pulmonary disease (COPD, 140 patients, 40.6%), asthma (13 patients, 3.8%), diabetes (68 patients, 19.7%), chronic kidney disease (CKD, 147 patients 42.6%), anaemia (39 patients, 11.3%), thrombopenia (130 patients, 37.7%), ascites (126 patients, 36.5%), pneumonia (151 patients, 43.8%), liver cirrhosis (110 patients, 31.9%), and hepatic encephalopathy (HE, 111 patients, 32.2%). Regarding tumour burden, the T stage distribution was as follows: T1 in 39 patients (11.3%), T2 in 46 patients (13.3%), T3 in 53 patients (15.4%), and T4 in 207 patients (60%). The N1 stage was observed in 128 patients (37.1%), the M1 stage was observed in 66 patients (19.1%), portal vein invasion was observed in 144 patients (41.7%), and microvascular invasion was observed in 183 patients (53%). The distribution of patients by type of therapy administered was mainly as follows: 196 patients, TACE (56.8%); 112 patients, RT (32.5%); and 37 patients, RFA (10.7%). The demographic and disease characteristics of the patients are shown in Table 1 and Table 2.

**Table 2:** Baseline Characteristics of Patients with HCC Following Interventional Therapy in Overall, Training, and Validation Cohorts.

Variables	Level	Overall Data	Training Data	Validation Data	P-value
		(N = 345)	(N = 242)	(N = 103)	
Gender (%)	Male	277 (80.3)	194 (80.16)	83 (80.6)	0.996
	Female	68 (19.7)	48 (19.84)	20 (19.4)	
Readmission (%)	Yes	103 (29.9)	71 (29.3)	32 (31.1)	0.949
	No	242 (70.1)	171 (70.7)	71 (68.9)	
Smoking (%)	Yes	116 (33.6)	79 (32.6)	37 (35.9)	0.840
	No	229 (66.4)	163 (67.4)	66 (64.1)	
Alcohol (%)	Yes	115 (33.3)	83 (34.3)	32 (31.1)	0.844
	No	230 (66.7)	159 (65.7)	71 (68.9)	
HBV infection (%)	Yes	160 (46.4)	108 (44.6)	52 (50.5)	0.607
	No	185 (53.6)	134 (55.4)	51 (49.5)	
Hypertension (%)	Yes	161 (46.7)	116 (47.9%)	45 (43.7)	0.769
	No	184 (53.3)	126 (52.1)	58 (56.3)	
CHD (%)	Yes	67 (19.4)	43 (17.8)	24 (23.3)	0.493
	No	278 (80.6)	199 (82.2)	79 (76.7)	
HF (%)	Yes	53 (15.4)	38 (15.7)	15 (14.6)	0.964
	No	292 (84.6)	204 (84.3)	88 (85.4)	
TIA (%)	Yes	151 (43.8)	101 (41.7)	50 (48.5)	0.506
	No	194 (56.2)	141 (58.3)	53 (51.5)	
COPD (%)	Yes	140 (40.6)	96 (39.7)	44 (42.7)	0.869
	No	205 (59.4)	146 (63.3)	59 (57.3)	
Asthma (%)	Yes	13 (3.8)	8 (3.3)	5 (4.9)	0.734
	No	332 (96.2)	234 (96.7)	98 (95.1)	
Diabetes (%)	Yes	68 (19.7)	45 (18.6)	23 (22.3)	0.727
	No	277 (80.3)	197 (81.4)	80 (77.7)	
CKD (%)	Yes	147 (42.6)	141 (58.3)	46 (44.7)	0.881
	No	198 (57.4)	101 (41.7)	57 (55.3)	
Anemia (%)	Yes	39 (11.3)	29 (11.9)	10 (9.7)	0.829
	No	306 (88.7)	213 (88.1)	93 (90.3)	
Thrombopenia (%)	Yes	130 (37.7)	99 (40.9)	31 (30.1)	0.165
	No	215 (62.3)	143 (59.1)	72 (69.9)	
Ascites (%)	Yes	126 (36.5)	83 (34.3)	43 (41.7)	0.421
	No	219 (63.5)	159 (65.7)	60 (58.3)	
Pneumonia (%)	Yes	151 (43.8)	92 (38.0)	59 (57.3)	0.004
	No	194 (56.2)	150 (62.0)	44 (42.7)	
Liver cirrhosis (%)	Yes	110 (31.9)	74 (30.6)	36 (35.0)	0.727
	No	235 (68.1)	168 (69.4)	67 (65.0)	
HE (%)	Yes	111 (32.2)	73 (30.2)	38 (36.9)	0.472
	No	234 (67.8)	169 (69.8)	65 (63.1)	
Portal Vein invasion (%)	Yes	144 (41.7)	94 (38.8)	50 (48.5)	0.247
	No	201 (58.3)	148 (61.2)	53 (51.5)	
Microvascular invasion (%)	Yes	183 (53.0)	128 (52.9)	55 (53.4)	0.996
	No	162 (47.0)	114 (47.1)	48 (46.6)	
T stage (%)	T1	39 (11.3)	31 (12.8)	8 (7.8)	0.483
	T2	46 (13.3)	37 (15.3)	9 (8.7)	
	T3	53 (15.4)	34 (14.0)	19 (18.4)	
	T4	207 (60.0)	140 (57.9)	67 (65.1)	
N Stage (%)	N0	217 (62.9)	160 (66.1)	57 (55.3)	0.165
	N1	128 (37.1)	82 (33.9)	46 (44.7)	
M Stage (%)	M0	279 (80.9)	194 (80.2)	85 (82.5)	0.878
	M1	66 (19.1)	48 (19.8)	18 (17.5)	
Therapy type (%)	TACE	196 (56.8)	132 (54.5)	64 (62.1)	0.788
	RT	112 (32.5)	83 (34.3)	29 (28.2)	
	RFA	37 (10.7)	27 (11.2)	10 (9.7)	

### Variable selection

To identify the most relevant predictors of in-hospital mortality among patients with HCC following interventional therapy, we employed LASSO regression. LASSO regression is particularly effective in handling datasets with numerous predictors and potential multicollinearity, as it performs both variable selection and regularization to enhance the prediction accuracy and interpretability of the statistical model.

**Figure 2:**
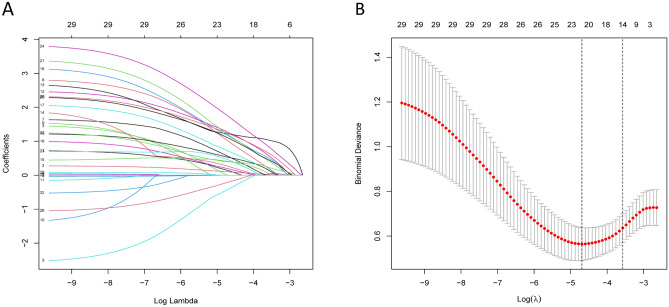
Demographic and clinical feature selection using LASSO binary logistic regression model.

Using the training dataset, we initially considered 29 potential predictors, including demographic factors, comorbidities, and clinical parameters. The LASSO regression model identified 21 significant predictors with non-zero coefficients (Fig. 2 A, B). These predictors included gender, LOS, smoking status, HBV status, hypertension, COPD status, asthma status, CKD status, diabetes, anemia, thrombopenia, ascites, pneumonia, liver cirrhosis, HE, portal vein invasion, microvascular invasion, N stage, T stage, therapy type and AFP. The optimal lambda value (λ.min) determined through cross-validation was 0.009, indicating the point at which the model’s performance is optimized by balancing model complexity and prediction accuracy (Table 3).

**Table 3:** Coefficients and lambda.min value of the LASSO regression.

Factors	Coefficients	Lambda.min
Gender	0.136	0.009
LOS	0.100	
Smoking	− 0.409	
HBV	0.553	
Hypertension	0.998	
COPD	1.259	
Asthma	1.213	
Diabetes	0.262	
CKD	0.407	
Anemia	1.158	
Thrombopenia	0.711	
Ascites	0.190	
Pneumonia	0.528	
Liver cirrhosis	1.227	
HE	1.188	
Portal Vein invasion	0.455	
Microvascular invasion	1.732	
T Stage	0.396	
N Stage	1.095	
Therapy type	− 0.202	
AFP	0.001	

### Multivariate logistic regression analysis

The 21 predictors identified by LASSO regression were further analysed using multivariate logistic regression to determine their independent associations with in-hospital mortality. This step was crucial to refine the model by identifying the most robust predictors while adjusting for potential confounders. The multivariate logistic regression analysis revealed that 10 variables—LOS, HBV status, hypertension, COPD status, anemia, thrombopenia, liver cirrhosis status, HE status, N stage, and microvascular invasion—were independently associated with in-hospital mortality (p < 0.05). These findings are detailed in Table 4 and underscore the significance of these ten factors as independent clinical predictors of hospitalization-related mortality in HCC patients following interventional therapy.

**Table 4:** Multivariate Logistic Regression Analysis of Variables in the Training Cohort.

Variables	Level	Multivariate		P-value
		OR	95% CI	
Gender	Male	Reference		0.562
	Female	1.62	0.30, 8.38	
LOS	No	Reference		0.007
	Yes	1.29	1.09, 1.60	
Smoking	No	Reference		0.099
	Yes	0.25	0.04, 1.17	
HBV	No	Reference		0.031
	Yes	5.58	1.28, 30.58	
Hypertension	No	Reference		0.003
	Yes	13.57	2.78, 97.71	
COPD	No	Reference		0.008
	Yes	8.80	1.96, 53.20	
Asthma	No	Reference		0.211
	Yes	4.10	0.44, 43.12	
Diabetes	No	Reference		0.589
	Yes	1.70	0.23, 11.60	
CKD	No	Reference		0.556
	Yes	1.62	0.31, 8.39	
Anemia	No	Reference		0.009
	Yes	13.54	2.09, 110.55	
Thrombopenia	No	Reference		0.039
	Yes	5.06	1.17, 27.17	
Ascites	No	Reference		0.341
	Yes	1.96	0.49, 8.18	
Pneumonia	No	Reference		0.262
	Yes	2.32	0.55, 11.03	
Liver cirrhosis	No	Reference		0.007
	Yes	8.28	1.90, 43.85	
HE	No	Reference		0.002
	Yes	14.63	3.07, 100.70	
Portal Vein invasion	No	Reference		0.358
	Yes	2.64	0.36, 24.20	
Microvascular invasion	No	Reference		0.000
	Yes	40.95	7.11, 409.75	
T Stage	T1	Reference		0.377
	T2	0.08	0.00, 23.73	
	T3	0.34	0.01, 51.31	
	T4	1.01	0.02, 132.97	
N Stage	N0	Reference		0.012
	N1	10.89	1.86, 84.85	
Therapy type	TACE	Reference		0.354
	RT	0.47	0.08, 2.20	
	RFA	0.22	0.01, 3.14	
AFP		1.00	0.997, 1.006	0.580

### Development of an individualized prediction model

**Figure 3:**
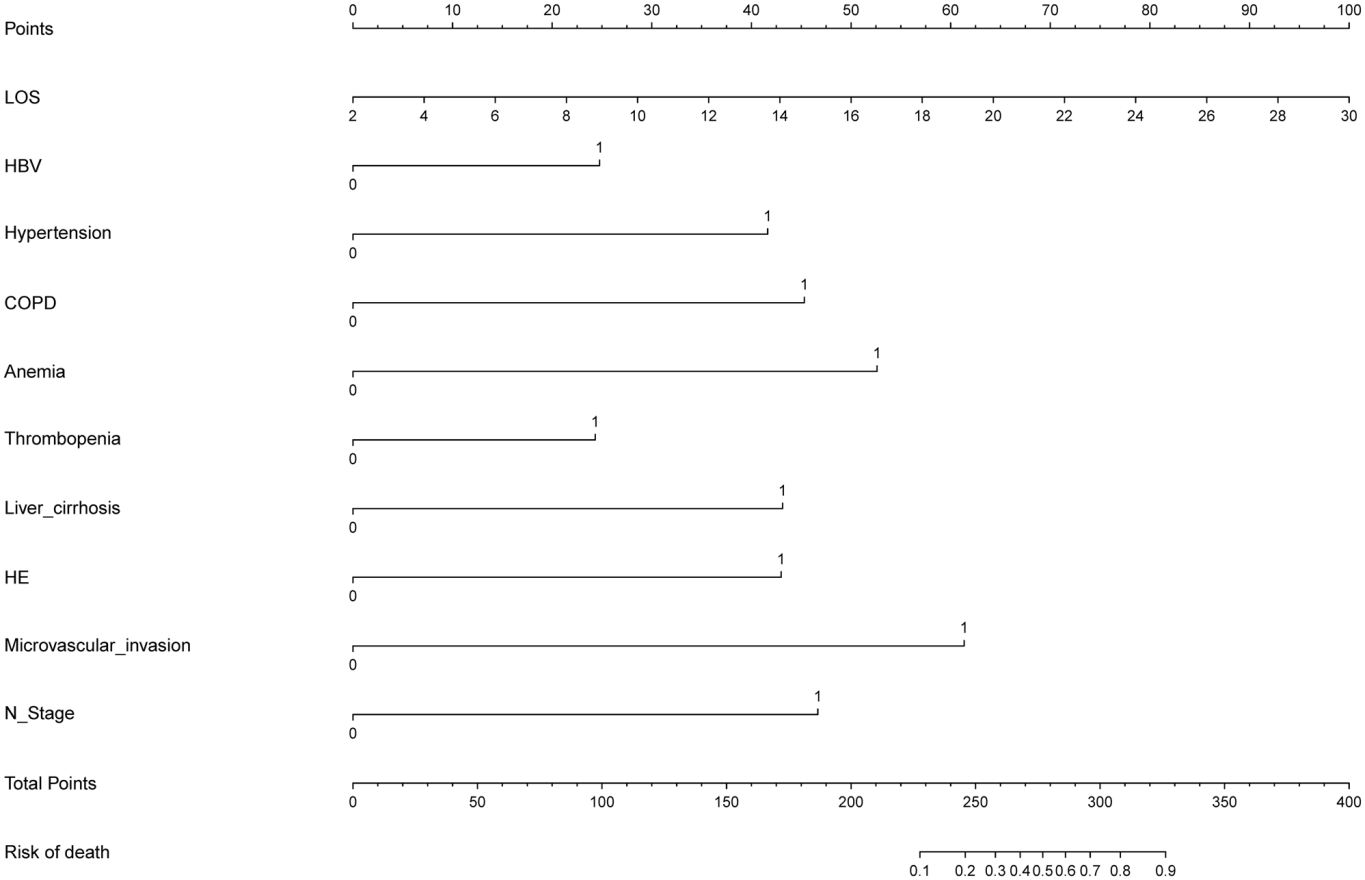
Nomogram for HCC patients.

Utilizing the ten variables identified by logistic regression analysis—LOS, HBV infection, hypertension, COPD, anemia, thrombopenia, liver cirrhosis, HE, N stage, and microvascular invasion—a nomogram was constructed to facilitate the prediction of mortality risk during hospitalization in HCC patients following interventional therapy (Fig. 3). The nomogram is a graphical representation of a predictive model that generates a numerical probability of a clinical event, such as in-hospital mortality. Each predictor variable is assigned a point value based on its contribution to the outcome. The points for all predictors are summed to generate a total score, which corresponds to the probability of in-hospital mortality. To use the nomogram, locate each predictor variable on its corresponding axis, draw a vertical line to the "Points" axis to determine the point value, sum the points for all predictors, and then locate the total points on the "Total Points" axis to find the predicted probability of mortality.

### Apparent performance of the in-hospital risk-of-death nomogram in the data cohort

**Figure 4:**
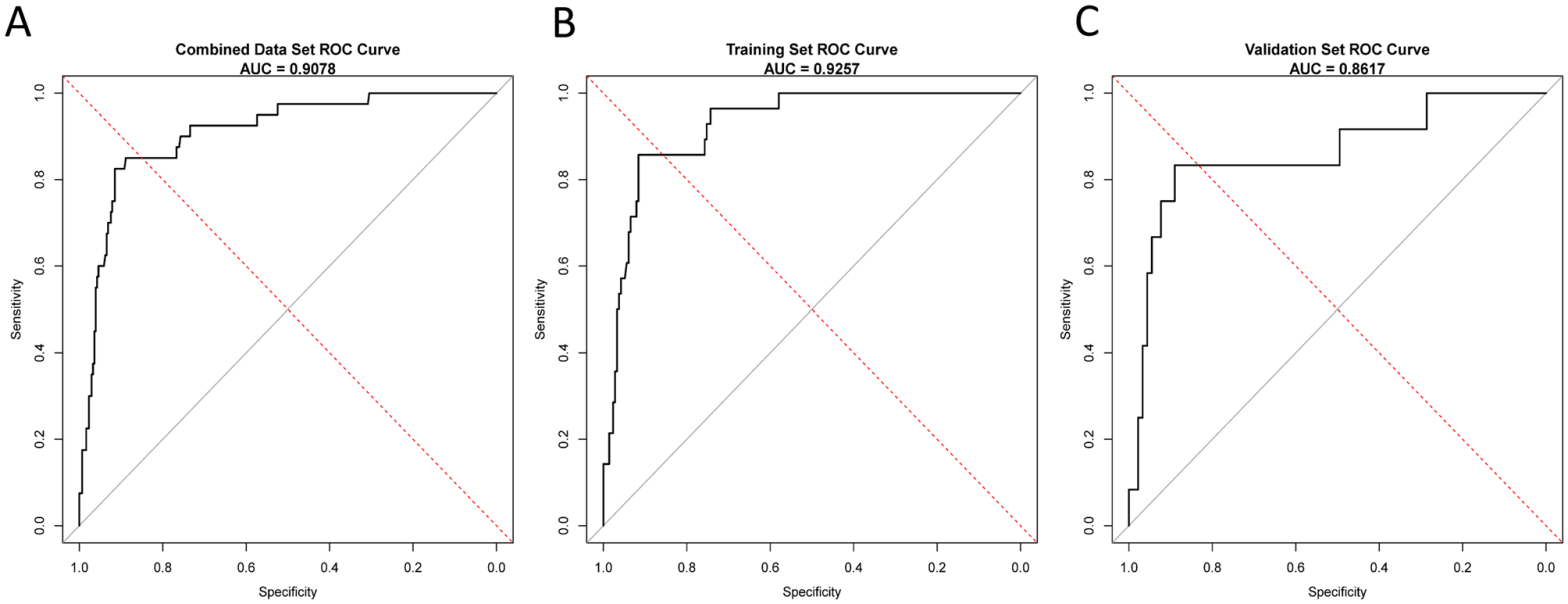
ROC curves for the training and validation set. overall set (**A**); training set (**B**); validation set (**C**).

Figure 4 illustrates the robust discriminative capability of the model as evidenced by the performance of the nomogram in predicting mortality during hospitalization. The effectiveness of our prediction nomogram was quantified by the C-index and AUC-ROC. In the training set, the AUC was 0.926 (95% CI = 0.883–0.968) (Fig. 4B), and in the validation set, the AUC was 0.862 (95% CI = 0.728–0.994) (Fig. 4C). For the overall dataset, the AUC was 0.908 (95% CI = 0.859–0.956) (Fig. 4A). These high AUC values indicate excellent discriminatory ability of the model. The bootstrapping validation further substantiated these findings, yielding a C-index of 0.908 for the overall dataset.

**Figure 5:**
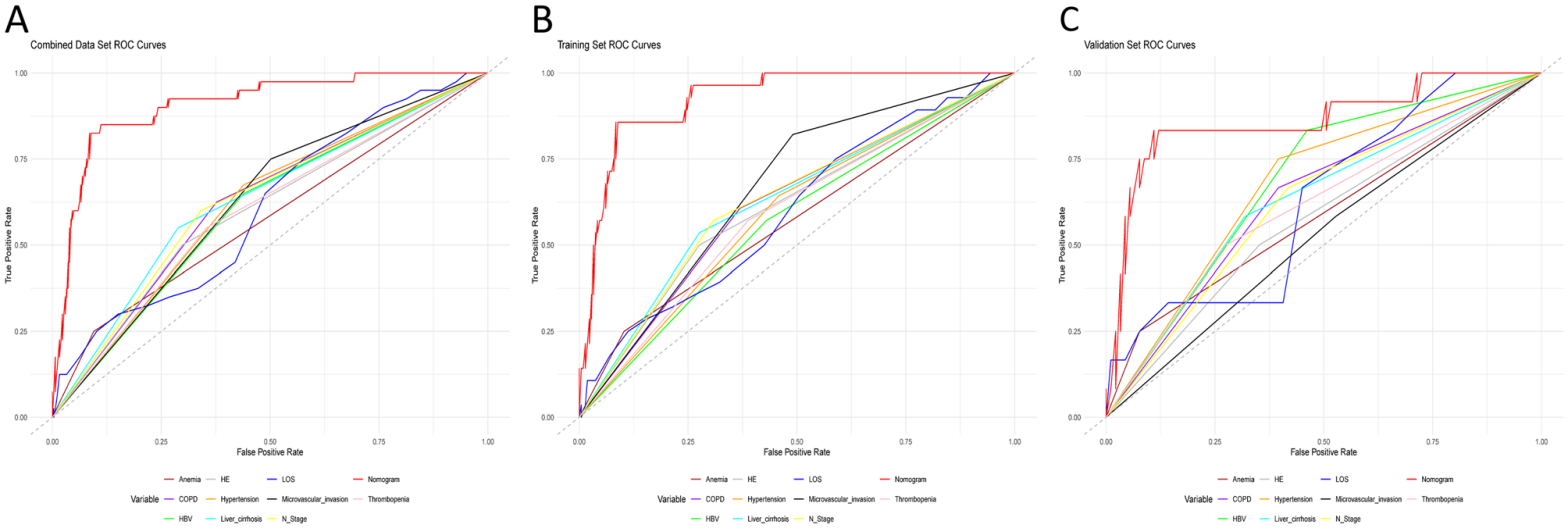
ROC curves for nomogram and independent predictor. overall set (**A**); training set (**B**); validation set (**C**).

Additionally, Fig. 5 presents the AUC for the nomogram compared to the AUC for each individual predictor included in the model. This comparison illustrates that the nomogram outperforms individual predictors, emphasizing its comprehensive predictive power. The AUC values for individual predictors in the overall dataset were: LOS (0.606), HBV (0.605), hypertension (0.618), COPD (0.624), anemia (0.577), thrombopenia (0.598), liver cirrhosis (0.631), HE (0.601), microvascular invasion (0.624), and N stage (0.630). The nomogram's AUC was significantly higher at 0.908 (Fig. 5A). In the training set, the AUC values for individual predictors were: LOS (0.600), HBV (0.571), hypertension (0.592), COPD (0.619), anemia (0.574), thrombopenia (0.592), liver cirrhosis (0.630), HE (0.612), microvascular invasion (0.665), and N stage (0.632). The nomogram's AUC in the training set was 0.926 (Fig. 5B). In the validation set, the AUC values for individual predictors were: LOS (0.629), HBV (0.686), hypertension (0.677), COPD (0.636), anemia (0.587), thrombopenia (0.613), liver cirrhosis (0.632), HE (0.574), microvascular invasion (0.528), and N stage (0.625). The nomogram's AUC in the validation set was 0.862 (Fig. 5C). Specific details of each model are provided in Table 5.

**Table 5:** AUC of overall dataset, training dataset and validation dataset.

Variable	Overall dataset		Training dataset		Validation dataset	
	AUC	95% CI	AUC	95% CI	AUC	95% CI
Nomogram	0.908	0.859–0.956	0.926	0.883, 0.968	0.862	0.728, 0.994
LOS	0.606	0.514–0.697	0.600	0.489–0.712	0.629	0.466–0.792
HBV	0.605	0.525–0.685	0.571	0.472–0.669	0.686	0.564–0.807
Hypertension	0.618	0.539–0.696	0.592	0.496–0.689	0.677	0.539–0.815
COPD	0.624	0.543–0.705	0.619	0.521–0.717	0.636	0.487–0.784
Anemia	0.577	0.507–0.647	0.574	0.489–0.658	0.587	0.455–0.717
Thrombopenia	0.598	0.515–0.681	0.592	0.493–0.691	0.613	0.457–0.767
Liver cirrhosis	0.631	0.548–0.713	0.630	0.531–0.729	0.632	0.478–0.786
HE	0.601	0.518–0.683	0.612	0.513–0.711	0.574	0.418–0.730
Microvascular invasion	0.624	0.550–0.698	0.665	0.586–0.745	0.528	0.373–0.682
N Stage	0.630	0.548–0.711	0.632	0.533–0.729	0.625	0.476–0.773

### Calibration of the predictive model

**Figure 6:**
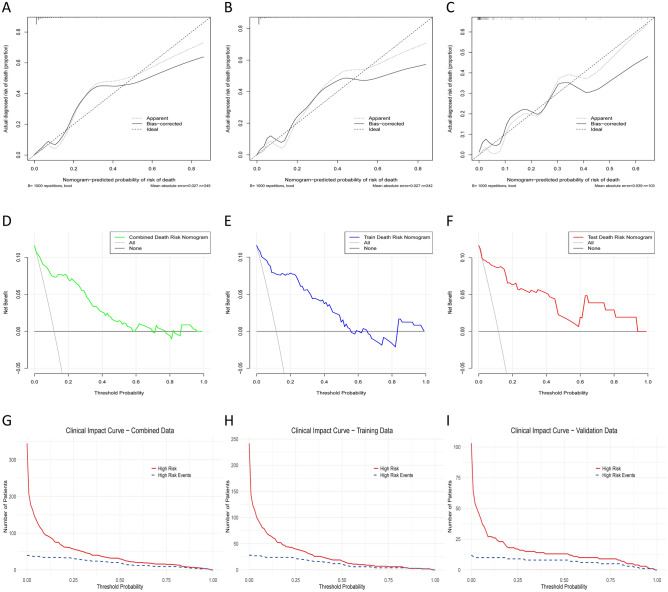
Calibration diagram. overall dataset (**A**); training dataset (**B**); validation dataset (**C**). Decision curve. overall dataset (**D**); training dataset (**E**); validation dataset (**F**). Clinical impact curve. overall dataset (**G**); training dataset (**H**); validation dataset (**I**).

Calibration analysis assessed the accuracy of predicted probabilities against observed outcomes across the training (N = 242), validation (N = 103), and overall (N = 345) datasets. In the training dataset, the model showed good calibration with a mean absolute error (MAE) of 0.027 and a mean squared error (MSE) of 0.00209, with 90% of predictions within an absolute error of less than 6.9% (Fig. 6B). The validation dataset had a slightly higher MAE of 0.039 and an MSE of 0.00274, with 90% of predictions within 7.1% of actual outcomes (Fig. 6C). The overall dataset demonstrated similar calibration to the training set, with an MAE of 0.027, an MSE of 0.00236, and 90% of predictions within 9.2% of actual outcomes (Fig. 6A).

### Clinical use

The Decision Curve Analysis (DCA) of the nomogram assessed the clinical utility for predicting in-hospital death. The nomogram was beneficial for threshold probabilities of 1%-25% in the training set, 1%-15% in the validation set, and 1%-25% in the overall dataset. In the training set, the nomogram provided a higher net benefit than treating all or no patients within a threshold range of 0.01 to 0.20, with the greatest benefit at a threshold probability of 0.10 (Fig. 6E). In the validation set, the nomogram demonstrated superior net benefit within a threshold range of 0.01 to 0.15, with the maximum net benefit at approximately 0.05 (Fig. 6F). The overall dataset DCA showed consistent net benefit across a threshold range of 0.01 to 0.25, with the highest benefit at around 0.10 (Fig. 6D). These findings highlight the model's effectiveness in guiding clinical decision-making and optimizing patient outcomes.

The Clinical Impact Curve (CIC) illustrated the clinical impact of the nomogram by showing the number of high-risk patients and high-risk events at various threshold probabilities. In the training set, the number of high-risk patients decreased from 242 at a threshold of 0.00 to 20 at a threshold of 0.50, while high-risk events ranged from 28 to 12 (Fig. 6H). The validation set showed similar trends, with high-risk patients decreasing from 103 to 8 and high-risk events from 12 to 3 (Fig. 6I). The overall dataset CIC showed high-risk patients decreasing from 345 to 28 and high-risk events from 40 to 15 (Fig. 6G). This analysis supports the nomogram's clinical utility and reliability, accurately identifying high-risk patients and predicting high-risk events.

### Risk groups

Predicted probabilities of in-hospital mortality were calculated for all patients using the nomogram. A threshold of 0.20 was selected to define high-risk and low-risk groups based on the Youden Index and validated using the testing dataset. Patients with probabilities above 0.20 were classified as high-risk, while those below 0.20 were low-risk. This classification resulted in 89 high-risk (25.8%) and 256 low-risk (74.2%) patients. Observed in-hospital mortality rates were 28.1% for high-risk and 6.3% for low-risk groups, highlighting the nomogram's effectiveness in identifying at-risk patients.

The risk stratification approach was evaluated using the AUC-ROC and decision curve analysis (DCA). The AUC-ROC for the risk groups was 0.844 (95% CI = 0.787–0.901), indicating good discriminatory ability. DCA showed a higher net benefit for the risk-based approach compared to treating all or no patients across various threshold probabilities, confirming its clinical utility.

## Discussion

Patients with HCC who cannot undergo surgical resection often present with varying degrees of comorbidity burdens^20,21^. These include multimorbidity disease related to the liver tumour itself as well as nonhepatic comorbidities, such as hypertension, diabetes, and renal failure^21,22^. In our study, a diverse range of comorbid conditions accompanying HCC were exhibited in substantial proportions of patients. Specifically, 46.4% of the patients had HBV infection, 46.7% presented with hypertension, 40.6% had COPD, 37.7% had thrombocytopenia, 11.3% had anaemia, 31.9% had liver cirrhosis, and 32.2% manifested HE.

Notably, 46.1% of the HCC patients in our cohort were older than 65 years, suggesting a potential correlation between advanced age and increased comorbidity incidence. The increasing complexity of comorbidities with age underscores the need for a deeper understanding of how age-related factors contribute to the comorbidity burden in HCC patients^21,23,24^. This trend highlights the multifaceted challenges in managing HCC, particularly in the context of an ageing population.

The presence and type of these comorbidities are key factors affecting the adverse prognosis in HCC patients^22^. The variety and cumulative effect of these comorbidities can increase the risk of longer hospital stays, in-hospital mortality, and postoperative complications for HCC patients^25^. In our cohort of HCC patients following interventional therapy, deaths were recorded during hospitalization, corresponding to an in-hospital mortality rate of 11.59%. Logistic regression analysis revealed that the use of TACE, RT, or RFA as treatment modalities was not significantly associated with the risk of in-hospital mortality (*p* = 0.667). These findings suggest that the choice among these therapeutic interventions may not significantly influence immediate survival outcomes in hospitalized HCC patients, underscoring the need for further investigation into other factors influencing mortality. Additionally, the severity of these comorbidities plays a crucial role in determining the choice of treatment modality for HCC patients^26^.

Among the 40 patients who experienced in-hospital mortality, the causes of death were distributed as follows: 12 patients (30%) died of liver failure and hepatic encephalopathy, 6 patients (15%) died of sepsis, 2 patients (5%) died of pneumonia, 8 patients (20%) died of multi-organ failure, 6 patients (15%) died of acute respiratory distress syndrome (ARDS), 4 patients (10%) died of gastrointestinal hemorrhage, 1 patient (2.5%) died of diabetic ketoacidosis, and 1 patient (2.5%) died of hyperosmolar hyperglycemic state. The relationship between chronic comorbidities, advanced HCC, and interventional therapies is complex and multifaceted, significantly contributing to the increased risk of in-hospital mortality observed in this study. The majority of deaths from liver failure and hepatic encephalopathy were observed in patients with advanced HCC and significant liver cirrhosis. Interventional therapies such as TACE, RT, and RFA, while aimed at controlling tumor growth, can further compromise already impaired liver function, leading to decompensation and failure. Infections, including sepsis and pneumonia, were significant causes of mortality, particularly in patients with chronic conditions such as COPD, diabetes, and CKD, who are more susceptible to infections due to their compromised immune systems. Multi-organ failure, often a terminal event in patients with advanced cancer and multiple comorbidities, was a substantial cause of death, highlighting the need for meticulous monitoring and supportive care. ARDS, resulting from both direct lung injury and indirect causes, was a notable cause of death, especially in patients with pre-existing respiratory conditions. Gastrointestinal hemorrhage, often associated with portal hypertension in advanced liver disease, further increased mortality risk, particularly in patients with thrombopenia and anemia. Although less common, diabetic ketoacidosis and hyperosmolar hyperglycemic state were fatal complications in two patients, underscoring the metabolic vulnerability of patients with diabetes undergoing stressful procedures.

These findings emphasize the significant impact of chronic comorbidities on the outcomes of HCC patients undergoing interventional therapy. The presence of multiple chronic conditions exacerbates the risks associated with advanced cancer and invasive treatments, leading to higher in-hospital mortality rates. This comprehensive understanding of mortality causes underscores the necessity for integrated, multidisciplinary care approaches to address both the oncological and comorbid aspects of patient health, ultimately aiming to improve patient outcomes and quality of care.

Currently, there is a lack of comprehensive research on the impact of multiple comorbidities on the short-term prognosis of hospitalized HCC patients after interventional treatments. To address this gap, we conducted a retrospective exploratory study and selected 345 patients with HCC who were hospitalized for interventional treatments. Our focus was on assessing and identifying risk factors that could increase the likelihood of in-hospital mortality, and we developed a comprehensive nomogram integrating comorbid diseases (including HBV, hypertension, COPD, anaemia, thrombopenia, liver cirrhosis, and HE), LOS, N stage, and microvascular invasion. This tool demonstrated remarkable proficiency in predicting the risk of in-hospital death among HCC patients receiving interventional therapy. The excellent discrimination and calibration of the nomogram, coupled with DCA, underscore its potential clinical utility. Existing models for predicting outcomes in HCC primarily focus on tumour characteristics and liver function^11,27,28^. Our study adds a novel dimension by incorporating multimodal diseases, which are often overlooked. This approach aligns with growing evidence suggesting that comorbid conditions significantly impact cancer prognosis and treatment responses^4,20,29–31^. Unlike the previous models for predicting admission and functional decline^9^, our nomogram offers a more holistic view of patient health status to predict hospitalization death. The inclusion of multimodal diseases in our nomogram reflects the complex interplay between HCC and systemic health. Conditions such as HBV infection and liver cirrhosis directly impact liver cancer progression^29^, while comorbidities such as hypertension and COPD may complicate patient management during interventional therapies^32,33^. Our model suggests the need for a more integrated approach in treating HCC, where comorbidities are not mere footnotes but are central to planning and prognosis. The application of this nomogram in clinical settings could revolutionize patient assessment and personalized care. By accurately predicting in-hospital mortality risks, clinicians can make more informed decisions regarding intervention strategies, potentially improving survival outcomes and patient quality of life.

### Limitations

The interpretation of this study's findings must consider both its strengths and limitations. One notable strength is the extensive dataset, comprising a large cohort of HCC patients who underwent palliative locoregional therapy at a reputable university teaching hospital, ensuring high diagnostic accuracy. However, several limitations warrant attention. As a retrospective cross-sectional study, it captures only a single point in time, precluding longitudinal follow-up and limiting our ability to establish causal relationships and monitor long-term treatment effects. Additionally, the specificity of the patient population and clinical setting may restrict the generalizability of our findings to broader contexts, necessitating external validation in diverse cohorts. The reliance on routinely collected hospital administrative data might lead to the underreporting of comorbidities, resulting in a not common and heterogeneous population with conditions such as cirrhosis, potentially underestimating their impact on mortality risk. Furthermore, the predictive model was developed using single-center data, underscoring the need for validation across multiple centers and varied demographic and clinical settings. Prospective studies and multicenter validations are planned to further verify and refine the predictive model, thereby assessing its generalizability and accuracy across different patient populations.

## Conclusion

Our study emphasizes the importance of considering multimorbidity in the prognosis of HCC patients after interventional therapy and introduces a novel tool for predicting the risk of in-hospital death. This nomogram shows potential for enhancing patient-specific management strategies, ultimately contributing to improved outcomes in HCC patients.

### Supplementary Information

Supplementary Information.

## Data Availability

The datasets generated or analyzed during the current study are available from the corresponding author on reasonable request.
